# Impact of timing on in-patient outcomes of complete repair of tetralogy of Fallot in infancy: an analysis of the United States National Inpatient 2005–2011 database

**DOI:** 10.1186/s12872-019-0999-1

**Published:** 2019-02-26

**Authors:** Shihai Yang, Linlin Wen, Shuguang Tao, Jiangrong Gu, Jiangang Han, Junping Yao, Jianming Wang

**Affiliations:** Department of cardiac surgery, Children’s hospital in Hebei province, 133 Jianhua Nan Avenue, Shijiazhuang, 050200 Hebei province China

**Keywords:** Congenital heart disease, National Inpatient Sample, Neonate, Tetralogy of Fallot

## Abstract

**Background:**

This study aimed to investigate whether age at complete repair of tetralogy of Fallot (TOF) impacts postoperative morbidity and length of hospital stay in infants less than 365 days of age.

**Methods:**

The United States Nationwide Inpatient Sample was searched for infants 0–365 days of age that underwent complete repair of TOF between 2005 and 2011. Patients were categorized based on age at time of repair: 0–30 days; 31–90 days; 91–180 days; > 180 days.

**Results:**

A total of 1112 infants were included in the study. Multivariate analysis showed the risk of postoperative complications was 40% lower in infants ≥91 days old at the time of repair as compared to those ≤30 days old. In addition, children > 30 days old at the time of repair had a significantly shorter length of hospital stay than those aged ≤30 days. In the subgroup with elective repair, older age was associated with a shorter length of hospital stay as compared to those ≤30 days old at repair, while association between age at complete repair of TOF and postoperative complication was not significant among the groups after adjusting for confounders.

**Conclusions:**

In children < 1 year old, postoperative complications and length of hospital stay are affected by the timing of complete repair of TOF.

## Background

Tetralogy of Fallot (TOF) accounts for approximately 5 to 7% of all congenital cardiac conditions [1, 2]. As the most common form of cyanotic congenital heart disease, the prevalence of TOF is approximately 3.9 cases per 10,000 live births [1, 2]. The condition that was first described by Fallot in 1888 consists of anterior malalignment of the conoventricular septum [3]. The malalignment gives rise to the 4 classic features of the condition: 1) malalignment ventricular septal defect (VSD); 2) overriding of the aorta; 3) right ventricular outflow tract (RVOT) obstructions; and 4) right ventricular hypertrophy [3]. The degree of RVOT obstructions determines the degree of cyanosis at birth and can range from severely cyanotic to asymptomatic [3].

For decades there has been lots of discussion regarding the best time for surgical repair of TOF. The reasons for the debate include concern of higher morbidity and mortality at an earlier age of surgery, and if earlier surgery results in more normal development of the heart and lungs [3–5]. The traditional method for asymptomatic infants has been to wait until symptoms develop or the children are older to decrease the morbidity and mortality associated with operating on infants, and surgery at a younger age has been associated with an increased need for transannular patching at a later date [6]. For infants who are cyanotic, the decision revolves around a palliative shunt procedure and then complete repair at a later date or initial complete repair [7, 8].

With advances in surgical techniques and postoperative intensive care unit (ICU) management there has been an overall trend to perform primary complete repair on younger infants, regardless of the presence of symptoms or not [3, 7, 9–11]. There is also considerable variability between centers in the timing of repair [3, 12]. Many studies have been done examining the outcomes of early compared to late repair. The majority of studies have found that complete primary repair in infancy is associated with low morbidity and mortality and overall good outcomes [7, 9, 11, 13–17]. On the other hand, some studies, including a recent meta-analysis and multicenter study, have reported that early surgery is associated with increased morbidity and mortality [12, 18, 19].

To this end, the purpose of this study was to use data from a nationwide hospital-based database to investigate whether age at complete repair of TOF impacts short-term outcomes, such as postoperative morbidity and length of hospital stay, in infants less than 365 days of age.

## Methods

### Data source

This was a population-based, retrospective observational study. The Nationwide Inpatient Sample (NIS) is the largest all-payer United States inpatient care database [20]. It contains over one hundred clinical and non-clinical data elements from approximately 8 million hospital stays each year. The data elements include primary and secondary diagnoses, primary and secondary procedures, admission and discharge status, patient demographic characteristics, expected payment source, length of stay, and hospital characteristics. The most recent NIS database contains data from about 1050 hospitals from 44 States in the United States to approximate a 20% stratified sample of United States community hospitals as defined by the American Hospital Association. The NIS also provides statistical weights that allow estimates of national case volumes to be extrapolated. The NIS was developed as part of United States Healthcare Cost and Utilization Project (HCUP), which is sponsored by the Agency for Healthcare Research and Quality. Further information about the HCUP is available at their website (https://www.hcup-us.ahrq.gov). Because the NIS originally received signed informed consent from all included patients to participate in data collection for later evaluation, and patient data in the NIS database were subsequently de-identified, signed informed consent was waived for the present study. The present study obtained the certificate number, HCUP-71JWS18J9, and conforms to the data-use agreement for the NIS from the HCUP Project.

### Study population

Infants 0–365 days of age who underwent complete repair of TOF between 2005 and 2011 were identified in the HCUP-NIS database. Identification was based on an International Classification of Diseases, Ninth Revision (ICD-9) principle procedure code of 35.81. Then the study cohort was further categorized into 4 groups according to their actual age at complete repair of TOF: 0–30 days; 31–90 days; 91–180 days; > 180 days.

### Endpoints and outcome measures

The primary endpoints were length of hospital stay and postoperative complications.

Postoperative complications were defined by the following ICD-9 codes: cardiac complications and postoperative arrhythmia: 997.1, 998.0; respiratory complications and pneumonia: 997.3; infection/sepsis: 998.5, 995.9; other complications: 997.2, 997.4–997.9, 998.2–998.4, 998.6–998.9, 997.0, 998.1; re-operation 35.95; and died in hospital.

### Covariates

Patient characteristics examined included age at repair, sex, race/ethnicity, household income, insurance status (primary payer), and admission type (elective admission or not). Co-morbid conditions studied included chromosomal anomalies (ICD-9: 655.10, 758).

Related interventions analyzed included heart catheterization (ICD-9 procedure codes: 37.21–37.23, 89.64), use of extracorporeal membrane oxygenation (ECMO) (ICD-9: 39.65), pacemaker/defibrillator implantation (ICD-9: 37.7, 37.8, 37.94–37.98, 39.64), thoracentesis/pericardiocentesis/pluerodesis/chest tube placement/pericardiotomy (ICD-9: 34.91, 37.0, 34.92, 34.04, 37.12), gastrostomy tube insertion (ICD-9: 43.0, 43.1), blood transfusion (ICD-9: 99.0) and systemic to pulmonary artery shunt (ICD-9: 39.0). Hospital-related characteristics studied included hospital location (rural or urban), bed size, and annual caseload of TOF repair.

### Statistical analysis

Age at the surgery was calculated by the summing of the age at admission and the number of days from admission to the surgery. For patients without information of the number of days from admission to the surgery were impute with the mean of number of days from admission to the surgery for total population. Age was divided into 4 groups for analysis: ≤ 30, 30–90, 91–180, and > 180 days. Continuous variables were presented as the mean ± standard error, and were tested by analysis of variance (ANOVA) to determine differences between age groups; categorical variables were presented as counts and weighted percentages, and differences between age groups were tested by chi-square test.

Univariate and multivariate regression analyses were performed to examine the association between length of stay, postoperative complications, and age groups. Multivariate regression analyses were performed by adjusting for variables with values of *P* < 0.05 in the univariate analysis. Additionally, subgroup analysis was performed to determine the association between length of stay, postoperative complications, and age groups for children who were admitted electively. This was done to exclude symptomatic patients requiring emergent surgery, and thus focus only on the optimal timing of complete repair. Weighted samples, stratum, cluster were used to produce national estimates for all analyses. Two-sided *P* values of < 0.05 were considered statistically significant. All statistical analyses were performed by SAS® version 9.4 (Windows NT version, SAS Institute, Inc., Cary, NC, USA).

## Results

There were 55,906,462 hospitalizations recorded during the period from 2005 to 2011 in the HCUP-NIS database. Of these, 1112 had a principle procedure code of complete repair of TOF and were aged ≤1 year (365 days). The analytic sample size was equivalent to a population-based sample size of 5538 participants.

The baseline characteristics of the 1112 patients are shown in Table 1. The length of hospital stay was significantly longer for infants ≤30 days old at the time of repair (≤ 30 days old: 28.9 days, 31–90 days old: 16.4 days, 91–180 days old: 10.5 days, and > 180 days old: 10.3 days). A higher proportion of infants ≤30 days old at repair were male (≤ 30 days old: 59.7%, 31–90 days old: 61.8%, 91–180 days old: 60.9%, and > 180 days old: 52.9%); White (≤ 30 days old: 68.2%, 31–90 days old: 63.9%, 91–180 days old: 54.8%, and > 180 days old: 50.7%); non-electively admitted (≤ 30 days old: 90.3%, 31–90 days old: 39.7%, 91–180 days old: 20.0%, and > 180 days old: 21.0%); had received heart catheterization (≤ 30 days old: 18.9%, 31–90 days old: 4.9%, 91–180 days old: 4.2%, and > 180 days old: 5.4%); had received gastrostomy tube insertion (≤ 30 days old: 2.9%, 31–90 days old: 0.5%, 91–180 days old: 0%, and > 180 days old: 1.2%). On the other hand, a lower proportion of infants ≤30 days old at repair had chromosomal anomalies (≤ 30 days old: 4.1%, 31–90 days: 5.7% old, 91–180 days old: 10.4%, and > 180 days old: 13.4%) (Table 1).

**Table 1:** Baseline characteristic of study population, HCUP-NIS 2005–2011 (Unweighted *n* = 1112; Weighted *n* = 5538)

	Age: ≤ 30 days	Age: 31–90 days	Age: 91–180 days	Age: > 180 days	*P*-value^a^
	*n* = 101	*n* = 198	*n* = 449	*n* = 364	
Demographic data					
Age (days)	9.7 ± 0.7	64.4 ± 0.9	134.8 ± 1.2	245.0 ± 2.9	< 0.01^b^*
Length of stay (days)	28.9 ± 3.3	16.4 ± 1.7	10.5 ± 0.5	10.3 ± 0.6	< 0.01^b^*
Sex					
Male	60 (59.66%)	122 (61.76%)	274 (60.99%)	192 (52.94%)	0.04*
Female	41 (40.34%)	76 (38.24%)	175 (39.01%)	172 (47.06%)	
Race/ethnicity					
White	48 (68.22%)	93 (63.86%)	164 (54.77%)	134 (50.72%)	0.03*
Non-White	23 (31.78%)	53 (36.14%)	135 (45.23%)	129 (49.28%)	
Household income					
Q1	23 (22.74%)	46 (23.63%)	107 (23.94%)	96 (26.91%)	0.18
Q2	27 (28.04%)	45 (23.17%)	109 (24.35%)	94 (26.58%)	
Q3	22 (22.35%)	42 (21.62%)	125 (28.08%)	93 (26.15%)	
Q4	26 (26.87%)	62 (31.58%)	105 (23.64%)	73 (20.36%)	
Insurance status					
Medicare/Medicaid	49 (48.48%)	72 (36.29%)	193 (42.69%)	189 (52.05%)	0.01*
Private including HMO	47 (46.7%)	115 (58.24%)	227 (51.02%)	154 (42.36%)	
elf-pay/no charge/other	5 (4.82%)	11 (5.47%)	28 (6.3%)	21 (5.58%)	
Admission type					
Elective	10 (9.71%)	120 (60.35%)	355 (79.97%)	285 (78.99%)	< 0.01*
Non-elective	91 (90.29%)	77 (39.65%)	90 (20.03%)	77 (21.01%)	
Chromosomal anomalies	4 (4.13%)	11 (5.72%)	47 (10.41%)	48 (13.4%)	0.01*
Related Interventions					
Heart catheterization	19 (18.92%)	10 (4.92%)	19 (4.19%)	20 (5.37%)	< 0.01*
ECMO	4 (4.12%)	1 (0.52%)	0 (0%)	1 (0.28%)	–
Implantation of pacemaker / defibrillator	9 (8.97%)	16 (7.67%)	29 (6.38%)	18 (4.74%)	0.31
Thoracentesis, pericardiocentesis, pluerodesis, chest tube, pericardiotomy	7 (6.74%)	10 (5.16%)	39 (8.71%)	33 (9.01%)	0.36
Gastrostomy tube insertion	2 (2.06%)	5 (2.53%)	1 (0.22%)	2 (0.59%)	0.04*
Blood transfusion	31 (30.62%)	68 (34.18%)	140 (31.01%)	126 (34.73%)	0.69
Systemic to pulmonary artery shunt	3 (2.99%)	1 (0.48%)	0 (0%)	4 (1.19%)	–
Hospital characteristics					
Bed size of hospital					
Small	18 (17.72%)	32 (15.81%)	54 (11.88%)	49 (13.32%)	0.04*
Medium	15 (15.12%)	25 (12.96%)	94 (21.57%)	89 (25.53%)	
Large	68 (67.17%)	141 (71.23%)	301 (66.55%)	226 (61.15%)	
Location of hospital					
Rural	0 (0%)	1 (0.4%)	1 (0.21%)	1 (0.22%)	–
Urban	101 (100%)	197 (99.6%)	448 (99.79%)	363 (99.78%)	
Hospital caseload of complete repair of TOF	19.5 ± 1.6	20.9 ± 3.1	20.8 ± 2.1	19.8 ± 1.5	0.80^b^

Data presented as unweighted counts (weighted proportion) for categorical variables. Percentages may not add up to 100% due to missing data

Data presented as mean ± standard error (SE) for continuous variables

Q1: 0-25th percentile; Q2: 26th to 50th percentile; Q3: 51st to 75th percentile; Q4: 76th to 100th percentile

^a^Chi-square test; ^b^ANOVA

*Significant difference between groups, *P* < 0.05

- Test cannot be performed due to cells with zero

The frequencies of postoperative complications of infants who underwent complete repair of TOF are shown in Table 2. Infants aged ≤30 days at repair had a higher percentage of postoperative complications (≤ 30 days old: 40.8%, 31–90 days old: 26.9%, 91–180 days old: 23.7%, and > 180 days old: 23.4%. In addition, a larger percentage of infants aged ≤30 days at repair had cardiac complications/ postoperative arrhythmia, infections/sepsis, other complications, and died during hospitalization.

**Table 2:** Postoperative complications in children underwent complete repair of TOF

	Age: ≤ 30 days	Age: 31–90 days	Age: 91–180 days	Age: > 180 days	*P*-value^a^
	*n* = 101	*n* = 198	*n* = 449	*n* = 364	
Postoperative complications	41 (40.76%)	53 (26.9%)	107 (23.68%)	85 (23.4%)	< 0.01*
Cardiac complications/ postoperative arrhythmia	13 (13.23%)	32 (16.08%)	54 (12.01%)	30 (8.23%)	0.02*
Respiratory complications, pneumonia	5 (4.81%)	5 (2.47%)	21 (4.73%)	25 (6.91%)	0.09
Infection/sepsis	11 (10.75%)	8 (4.17%)	14 (3.06%)	6 (1.66%)	< 0.01*
Other complications	15 (14.88%)	19 (9.72%)	39 (8.56%)	41 (11.23%)	0.20
Re-operation	0 (0%)	2 (0.98%)	1 (0.23%)	3 (0.81%)	–
In-hospital mortality	10 (9.82%)	1 (0.51%)	3 (0.67%)	3 (0.85%)	< 0.01*

Data presented as unweighted counts (weighted proportion). Percentages may not add up to 100% due to missing data

Other complications include peripheral vascular complications, digestive system, urinary, nervous system complications, vascular complications of other vessels, accidental puncture or laceration during a procedure, disruption of the wound, persistent postoperative fistula, hemorrhage or hematoma complicating a procedure, foreign body accidentally left during a procedure, acute reaction to foreign substance accidentally left, other specified complications of procedures not elsewhere classified, and unspecified complication of procedure

^a^Chi-square test

*Significant difference between groups, *P* < 0.05

- Test cannot be performed due to cells with zero

The associations between length of stay, postoperative complications, and age at repair are shown in Table 3. Univariate analysis showed that children > 30 days old at the time of repair had a significantly shorter length of hospital stay than those aged ≤30 days (31–90 days old: − 12.5 days, 91–180 days old: − 18.4 days, and > 180 days old: − 18.6 days). However, after adjustment for insurance status, admission type, heart catheterization and gastrostomy tube insertion in the multivariate analysis, the result remained significant except for 31–90 days group (Table 3). When treated as continuous variable, older age at repair was significantly associated with slightly shorter length of hospital stay (β: − 0.02).

**Table 3:** Associations between age at complete repair of TOF and length of stay and postoperative complication

	Length of stay				Postoperative complication		
	Univariate	Multivariate	Multivariate^a^	Univariate	Multivariate	Multivariate^a^	
	β ± SE	β ± SE	β ± SE	OR (95% CI)	aOR (95% CI)	aOR (95% CI)	
Age at repair	**−0.05 ± 0.01**		− **0.02 ± 0.01**	**0.99 (0.99–1.00)**		**0.99 (0.99–1.00)**	
≤ 30 days	Reference	Reference		**–**	Reference	Reference	**–**
31–90 days	**−12.52 ± 3.31**	− 5.95 ± 2.98		**0.54 (0.34–0.84)**	0.66 (0.40–1.11)		
91–180 days	**− 18.38 ± 3.48**	**−8.72 ± 3.20**		**0.45 (0.31–0.66)**	**0.60 (0.39–0.92)**		
> 180 days	**−18.63 ± 3.54**	**−9.50 ± 3.39**			**0.44 (0.31–0.64)**	**0.60 (0.40–0.89)**	
Sex							
Male	Reference	**–**			Reference	**–**	**–**
Female	−0.18 ± 1.05				1.19 (0.92–1.53)		
Race/ethnicity							
White	Reference	**–**			Reference	**–**	**–**
Non-White	0.56 ± 1.90				1.29 (0.98–1.71)		
Insurance status							
Medicare/Medicaid	Reference	Reference	Reference	Reference	**–**	**–**	
Private including HMO	**−2.82 ± 1.04**	−1.78 ± 0.96	−1.95 ± 0.96	1.09 (0.85–1.39)			
Self-pay/no charge/other	−0.08 ± 2.7	0.83 ± 2.36	0.69 ± 2.36	0.88 (0.51–1.54)			
Admission type							
Elective	Reference	Reference	Reference	Reference	Reference		
Non-elective	**14.32 ± 2.72**	**10.17 ± 2.49**	**11.36 ± 2.52**	**1.56 (1.15–2.13)**	1.28 (0.91–1.78)	1.34 (0.99–1.83)	
Chromosomal anomalies	2.95 ± 2.17	**–**	**–**	0.95 (0.65–1.39)	–		
Heart catheterization	**15.72 ± 4.90**	**11.13 ± 4.37**	**11.81 ± 4.49**	**2.62 (1.67–4.10)**	**2.26 (1.41–3.62)**	**2.35 (1.48–3.73)**	
Gastrostomy tube insertion	**49.47 ± 11.45**	**42.61 ± 11.86**		**42.39 ± 11.31**	**0.36 (0.16–0.82)**	**0.28 (0.12–0.69)**	**0.27 (0.11–0.66)**
Bed size of hospital							
Small	Reference	**–**		**–**	Reference	Reference	Reference
Medium	0.61 ± 3.70				**0.62 (0.41–0.94)**	0.67 (0.44–1.03)	0.68 (0.43–1.07)
Large	0.60 ± 3.19				0.82 (0.53–1.27)	0.87 (0.57–1.33)	0.88 (0.56–1.36)

*β* estimate, *SE* standard error, *OR* odds ratio, *aOR* adjusted OR, *CI* confidence interval

Multivariate logistic regression was adjusted for variables significant in univariate analysis

Numbers in bold indicates statistically significant (*P* < 0.05)

- Test cannot be performed because variable was not significant in univariate analysis

^a^Age at repair was consider as continuous variable

Univariate analysis indicated that the risk of postoperative complications was significantly lower in children > 30 days old at the time of repair as compared to those ≤30 days old at repair (odds ratio [(OR]: 31–90 days old = 0.54, 91–180 days old = 0.45, and > 180 days old = 0.44). Multivariate analysis showed that the risk of postoperative complications was about 40% lower in children 91–180 days old and > 180 days old at the time of repair as compared to those ≤30 days old at repair (aOR: 91–180 days old = 0.60 and > 180 days old = 0.60) (Table 3). When treated as continuous variable, patients with older age at repair had lower risk of postoperative complications (aOR: − 0.99).

In the subgroup analysis of patients with elective admissions, older age was associated with a shorter length of hospital stay as compared to those ≤30 days old at repair (31–90 days old: − 2.94 days, 91–180 days old: − 3.95 days, and > 180 days old: − 4.29 days) in the multivariate analysis; however, the difference was not significant in the group 31–90 days old. Multivariate analysis indicated that older age groups had a 50 to 60% lower odds of postoperative complications as compared to infants ≤30 day old at repair (adjusted OR [aOR]: 31–90 days old = 0.43, 91–180 days old = 0.58, and > 180 days old = 0.58); however, the difference was not significant among the groups (Table 4).

**Table 4:** Associations between age at complete repair of TOF and length of stay and postoperative complication for children admitted electively (*n* = 770)

	Length of stay		Postoperative complication	
	Univariate	Multivariate^a^	Univariate	Multivariate^b^
	β ± SE	β ± SE	OR (95% CI)	aOR (95% CI)
Age at repair	−0.01 ± 0.01	−0.01 ± 0.005	0.99 (0.99–1.00)	1.00 (0.99–1.00)
≤ 30 days	0	0	1	1
31–90 days	−2.87 ± 1.87	−2.94 ± 1.89	**0.43 (0.19–0.97)**	0.43 (0.19–0.99)
91–180 days	− 3.48 ± 1.82	**−3.95 ± 1.77**	0.51 (0.25–1.02)	0.58 (0.28–1.18)
> 180 days	−3.43 ± 2.15	−**4.29 ± 2.06**	0.51 (0.25–1.03)	0.58 (0.27–1.22)

*β* estimate, *SE* standard error, *OR* odds ratio, *aOR* adjusted OR, *CI* confidence interval

Multivariate logistic regression was adjusted for variables significant in univariate analysis

Numbers in bold indicates statistically significant (*P* < 0.05)

^a^Multivariate analysis was adjusted for insurance status, gastrostomy tube insertion and bed size of hospital

^b^Multivariate analysis was adjusted for heart catheterization and bed size of hospital

## Discussion

The purpose of this study was to investigate whether the age of complete repair of TOF impacts short-term outcomes in infants less than 365 days of age. The results showed that the risk of postoperative complications was about 40% lower in infants ≥91 days old at the time of repair as compared to those ≤30 days old at repair after adjusting for the confounders.

In the subgroup of patients who underwent elective repair, the length of hospital stay of infants 91–180 days old at the time of surgery was about 3.5 days shorter compared to those who were ≤ 30 days old at repair after adjusting for the confounders. Additionally, in this subgroup the risk of postoperative complications was about 60% lower in children 31–90 days old at the time of repair as compared to those ≤30 days old after adjusting for confounders.

The majority of studies have indicated that complete repair of TOF can be performed in infancy with good outcomes. For example, Parry et al. (10) studied 42 acyanotic infants with a median age of 62 days who received complete repair of TOF between 1992 and 1999. They found that there were no deaths in-hospital or during a median follow-up period of 38 months; one child required reoperation for recurrent RVOT obstruction, and 2 required balloon angioplasty; and there were no cases of symptomatic pulmonary insufficiency. A recent study of 277 patients with TOF who received elective repair at 6 months of age or younger found no in hospital mortalities, and an 11.6% complication rate [13]. Furthermore, longer support times, lower weight, chromosomal abnormalities, and complications were associated with a significantly increased length of hospital stay. Another recent study compared outcomes in infants younger than 6 months and older than 6 months at the time of repair and found no difference in early clinical outcomes or reoperation rates [21].

Woldu et al. [17] studied the effect of the timing of surgery on growth in the first year of life. They found that 36 infants had neonatal TOF repair (< 28 days old) and 127 had repair at more than 28 days. Those who had neonatal repair were more likely to require a subsequent transannular patch, but had higher weight-for-age scores at 1 year.

A multicenter analysis study, however, has provided results different from those described above [19]. A study of 4698 patients between 1 day of age and 19 years who had complete repair of non-ductal-dependent TOF across a number of centers found that elective repair at < 30 days of age was associated with significantly higher in-hospital morbidity and mortality [19]. Furthermore, a meta-analysis published in 2017 that included 8 studies with 3858 patients, 19% of whom underwent neonatal repair, found that neonatal repair was associated with increased mortality, longer ICU stay, and longer total length of hospital stay [18].

There are a number of strengths, as well as limitations of this study. An important strength of the present study is that it includes the largest number of patients from all geographical regions in the United States, allowing for a significant analysis despite the relative rarity of the procedure. However, the diagnoses and procedures were identified based on the ICD coding system only, and coding errors and misclassifications might exist. In addition, coding limitations do not allow for differentiation of clinical subtleties during surgery or decisions by the individual surgeon. Although essential, the precise details of cardiac anatomy (degree of subplumonary stenosis, pulmonary valve annulus size, and coronary and pulmonary artery morphology), variations of surgical techniques and types, establishing feeding, cleft palate, and the duration of cardiopulmonary bypass and aortic cross-clamp time are not available in the database, and thus cannot be analyzed. Details of the clinical course, perioperative functional parameters, and medications prescribed during hospitalization at hospitalization are not provided in the database. Whether or not prior shunt palliation was performed could not be examined, and this study did not distinguish between cyanotic or asymptomatic disease. The database does not contain follow-up data after discharge, thus late mortality and other mid-term/long-term outcomes could not be evaluated.

Given the retrospective design, the possibility of residual measured or unmeasured confounding cannot be eliminated, as with any observational investigation. Nonetheless, the present study provides an overall picture of the hospital-related characteristics of children ≤1 year of age who underwent complete repair of TOF.

## Conclusions

In conclusion, the results showed that complete repair of TOF in infants > 90 days old was associated with lower postoperative complications and shorter length of hospital stay than in the neonates. In the subgroup with elective repair, older age was associated with a shorter length of hospital stay as compared to those ≤30 days old at repair, while association between age at complete repair of TOF and postoperative complication was not significant among the groups. Further well-designed prospective studies are warranted in order to evaluate the optimal timing for complete repair of TOF in infants.

## Abbreviations

ANOVA: Analysis of variance
ECMO: Extracorporeal membrane oxygenation
HCUP: Healthcare Cost and Utilization Project
ICU: Intensive care unit
NIS: Nationwide Inpatient Sample
RVOT: Right ventricular outflow tract
TOF: Tetralogy of Fallot
VSD: Ventricular septal defect
